# Triple-hit diffuse large B-cell lymphoma with choroidal and cavernous sinus involvement mimicking inflammatory and neuro-ophthalmic disease: case report

**DOI:** 10.3389/fopht.2026.1776913

**Published:** 2026-03-03

**Authors:** Christian Nieves-Rios, José Feneque González, Maria del Mar Rivera Rolon, Julio Rodriguez-Padilla, Victor M. Villegas, Luis Serrano

**Affiliations:** 1 Department of Ophthalmology, University of Puerto Rico School of Medicine, San Juan, Puerto Rico; 2 Department of Pathology, University of Puerto Rico School of Medicine, San Juan, Puerto Rico

**Keywords:** cavernous sinus syndrome, diffuse large B-cell lymphoma, ophthalmoplegia, serous retinal detachments, triple-hit

## Abstract

**Background:**

To describe our findings in a rare case of secondary triple-hit diffuse large B-cell lymphoma (DLBCL) with choroidal and central nervous system (CNS) involvement, presenting with bilateral serous retinal detachments (SRD) and ophthalmoplegia.

**Case presentation:**

A 44-year-old male presented with a 4-month history of bilateral vision loss, right-sided headaches radiating to the periorbital area, and oral numbness within the mental nerve distribution. Review of systems was notable for unintentional 25-pound weight loss and chronic back pain. Corrected visual acuities were 20/150 in the right eye and 20/50 in the left. External exam showed remarkable results for right-sided ptosis, diminished pupillary light response without afferent pupillary defect, and limitation of extraocular movements in all gazes. Fundus examination showed bilateral, multifocal SRD with associated increased choroidal thickness. Systemic workup showed right-sided prominence of the cavernous sinus, heterogeneous bone marrow signal throughout the spine, splenomegaly, retroperitoneal lymphadenopathy, and a hypodense hepatic lesion. Cerebrospinal fluid analysis revealed elevated white blood cell count and protein concentration. A lymph node biopsy revealed DLBCL with Bcl6, Bcl2, and c-Myc rearrangements. Treatment with combined intrathecal and systemic chemotherapy resulted in significant improvement in both systemic and ocular symptoms.

**Conclusion:**

This case underscores the importance of considering secondary lymphoma in patients presenting with bilateral SRD and neuro-ophthalmic deficits, even in the absence of known systemic malignancy. The combination of cavernous sinus syndrome with concomitant mandibular nerve involvement should prompt CNS and systemic evaluation for hematologic malignancy. Patients with triple-hit DLBCL phenotype may achieve dramatic visual recovery following modern targeted chemoimmunotherapy.

## Introduction

Diffuse large B-cell lymphoma (DLBCL) is the most common type of non-Hodgkin lymphoma (1, 2). It predominantly affects elderly patients, with a median age in the sixth decade (3), and occurs more frequently in white and male populations (1, 2). Based on cell of origin, DLBCL can be classified into germinal center B-cell-like and activated B-cell-like subtypes (4). Ophthalmic involvement may include the ocular adnexa (conjunctiva, orbit, eyelid, lacrimal gland) or intraocular compartments, such as the uvea (1).

Choroidal DLBCL may present as a primary ocular disease or as secondary ocular involvement in the context of systemic lymphoma with widespread dissemination (3, 5). Secondary choroidal lymphoma typically exhibits high-grade morphological features and is associated with more severe ocular manifestations (6). These cases are generally more likely to demonstrate bilateral ocular involvement, poorer visual outcomes, and extension to the iris or ciliary body (6). Ocular manifestations of systemic B-cell lymphoma include solitary masses in the posterior pole, adjacent serous retinal detachments (SRD), vitritis, retinal vasculitis, necrotizing retinitis, diffuse choroiditis (7), or focal uveal masses (8–10).

Despite advances in ocular imaging modalities, the diagnosis of secondary choroidal lymphomas remains challenging due to their rarity, diffuse distribution, ill-defined margins, and lack of intrinsic pigmentation (6, 11). This report describes a case of secondary triple-hit DLBCL with choroidal and central nervous system (CNS) involvement, presenting with bilateral SRD and ophthalmoplegia.

## Case presentation

A 44-year-old male with no significant past medical or ocular history presented with a 4-month history of progressive, bilateral vision loss and right-sided headaches radiating to the ipsilateral periorbital region. Review of systems was notable for a 3-month history of unintentional 25-pound weight loss and chronic back pain for 2 years. Social history was pertinent for a 28-year history of cigarette smoking. His family history was unremarkable. The patient was not taking any medications and denied recent travel or exposure to sick contacts.

**Figure 1:**
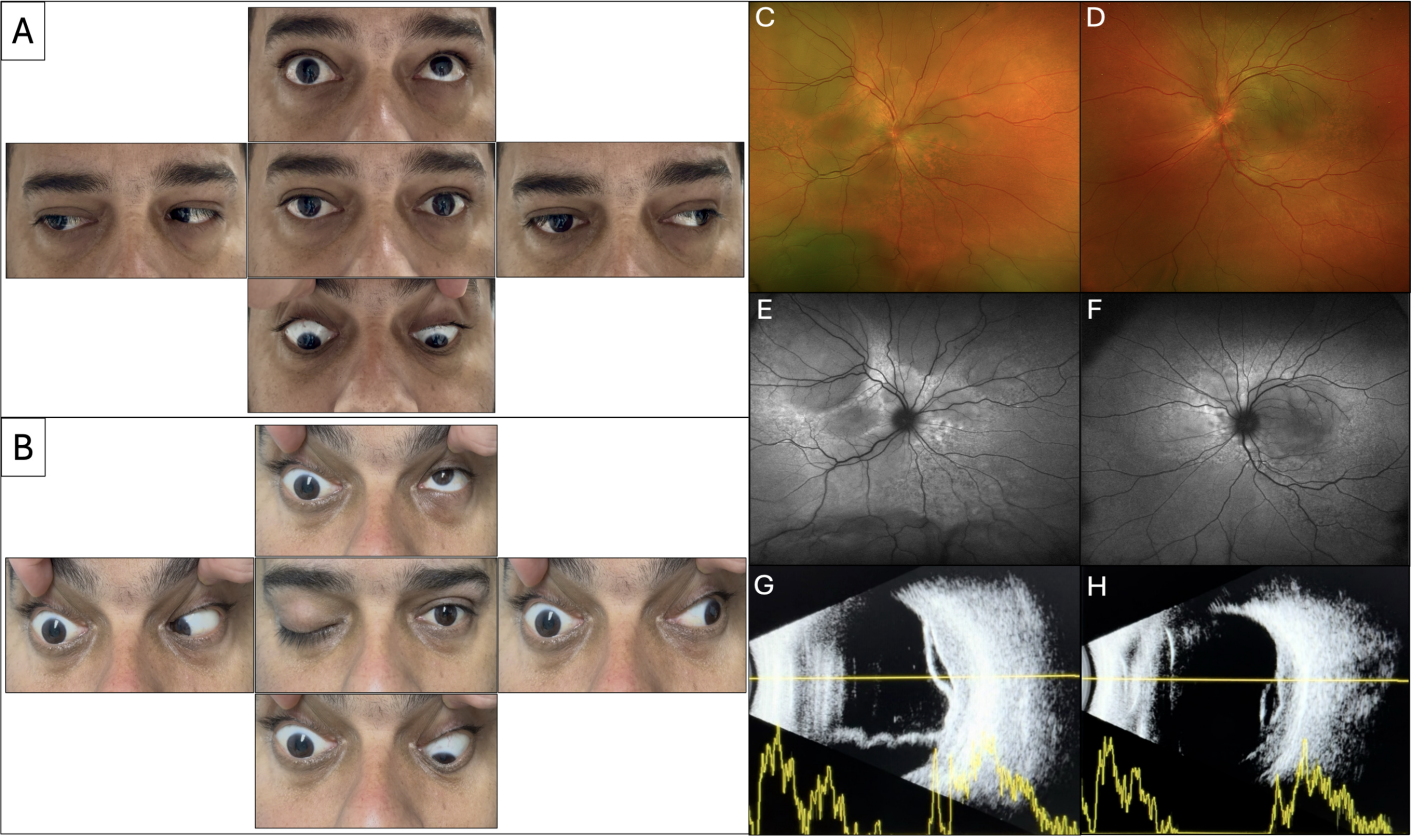
**(A)** Examination at presentation demonstrates limitation in extraocular movements in all directions of the right eye with associated mild ptosis. Movements in the left eye were complete in all gazes. No proptosis was noted. **(B)** Evaluation at the 3-day follow-up visit shows worsening ophthalmoplegia with progression to full ptosis on the right side. Extraocular movements in the left eye remain unchanged. **(C, D)** Fundus photographs demonstrate bilateral, multifocal SRD with subretinal fluid extension to the optic nerves, and patchy areas of hyper- and hypopigmentation in both the posterior pole and peripheral retina. **(E, F)** FAF revealed multifocal areas of hyper- and hypo-autofluorescence associated with SRD in both eyes. **(G, H)** B-scan ultrasonography confirmed the SRD and demonstrated areas of increased choroidal thickness. No evidence of posterior epibulbar extension or vitreous opacities was observed.

On examination, uncorrected visual acuities were counting fingers in the right eye and 20/400 in the left eye. Vision improved to 20/150 in the right eye and 20/50 in the left eye, with refractions of +7.75 −2.00 at 110° and +7.25 −0.50 at 100°, respectively. Intraocular pressures were normal bilaterally. The external examination revealed mild right-sided ptosis, with a superior margin-reflex distance of 3.5mm on the right and 4.5 mm on the left; no proptosis was identified. The right eye pupil exhibited a diminished pupillary light response, with a size of 6.0 mm in both darkness and light stimulus. The left pupil size was 4.0 mm in darkness, which decreased to 3.0 mm with light stimulus. No relative afferent pupillary defect was identified on reverse testing. Extraocular movements (EOM) were partially limited in all directions in the right eye, with no intorsion, and full in the left eye (**Figure 1A**). Forced duction testing showed a negative result.

Anterior segment examination was notable for mild conjunctival injection and chemosis in the right eye. There was no evidence of keratic precipitates, anterior chamber reaction, or vitreous inflammation. Fundus exam demonstrated bilateral, multifocal SRD with extension to the optic nerves, and patchy areas of hyper- and hypopigmentation (**Figures 1C, D**). Cranial nerve assessment demonstrated ipsilateral trigeminal hypoesthesia involving the mental nerve distribution and sparing of the maxillary nerve.

Fundus autofluorescence (FAF) revealed multifocal areas of hyper- and hypoautofluorescence associated with SRD in both eyes (**Figures 1E, F**), likely reflecting alternating areas of retinal pigment epithelium stress and atrophy secondary to choroidal infiltration and chronic SRD. B-scan ultrasonography confirmed the SRD and demonstrated areas of increased choroidal thickness measuring 4.6 mm in the right eye and 3.7 mm in the left eye at the maximal elevation. No evidence of posterior epibulbar extension or vitreous opacities was observed (**Figures 1G, H**). Other imaging modalities, including optical coherence tomography (OCT), fundus angiography (FA), and indocyanine green angiography (ICGA), were performed at presentation but were deemed unreliable because the patient was unable to properly fixate.

Outpatient laboratory tests ordered by his primary care provider revealed an erythrocyte sedimentation rate of 42 mm/h, a C-reactive protein level of 188 mg/L, a creatinine level of 1.49 mg/dL, a lactate dehydrogenase (LDH) level of 362 U/L, and positive IgG antibodies to herpes simplex viruses 1 and 2. Otherwise, the comprehensive metabolic panel, complete blood count, and thyroid-stimulating hormone were within normal limits.

Given the systemic symptoms, along with unilateral ophthalmoplegia, ptosis, and anisocoria in the setting of bilateral, multifocal SRD, the initial diagnostic impression was broad and included inflammatory, vascular, and neoplastic etiologies. Vogt–Koyanagi–Harada (VKH) disease, sympathetic ophthalmia, and central serous chorioretinopathy were considered. However, the absence of intraocular inflammation, integumentary or auditory findings, and any history of recent illness, trauma, or steroid use would be atypical for these entities. Ophthalmoplegia due to orbital inflammatory pseudotumor was also contemplated, as was cranial nerve palsy secondary to compressive lesions such as an aneurysm or malignancy (e.g., metastasis or lymphoma). The ipsilateral efferent pupillary defect suggested a compressive mechanism over the superficial parasympathetic fibers of the oculomotor nerve, while concomitant involvement of the trochlear and abducens nerves placed emphasis on lesions involving the cavernous sinus, superior orbital fissure, or orbital apex. Notably, the absence of an afferent pupillary defect reduced the likelihood of an orbital apex lesion. In addition, etiologies of increased intracranial pressure were considered, given the bilateral optic nerve changes. Ischemic optic neuropathy and optic neuritis were plausible yet lower in the differential given the patient’s demographics and the chorioretinal and systemic findings.

**Figure 2:**
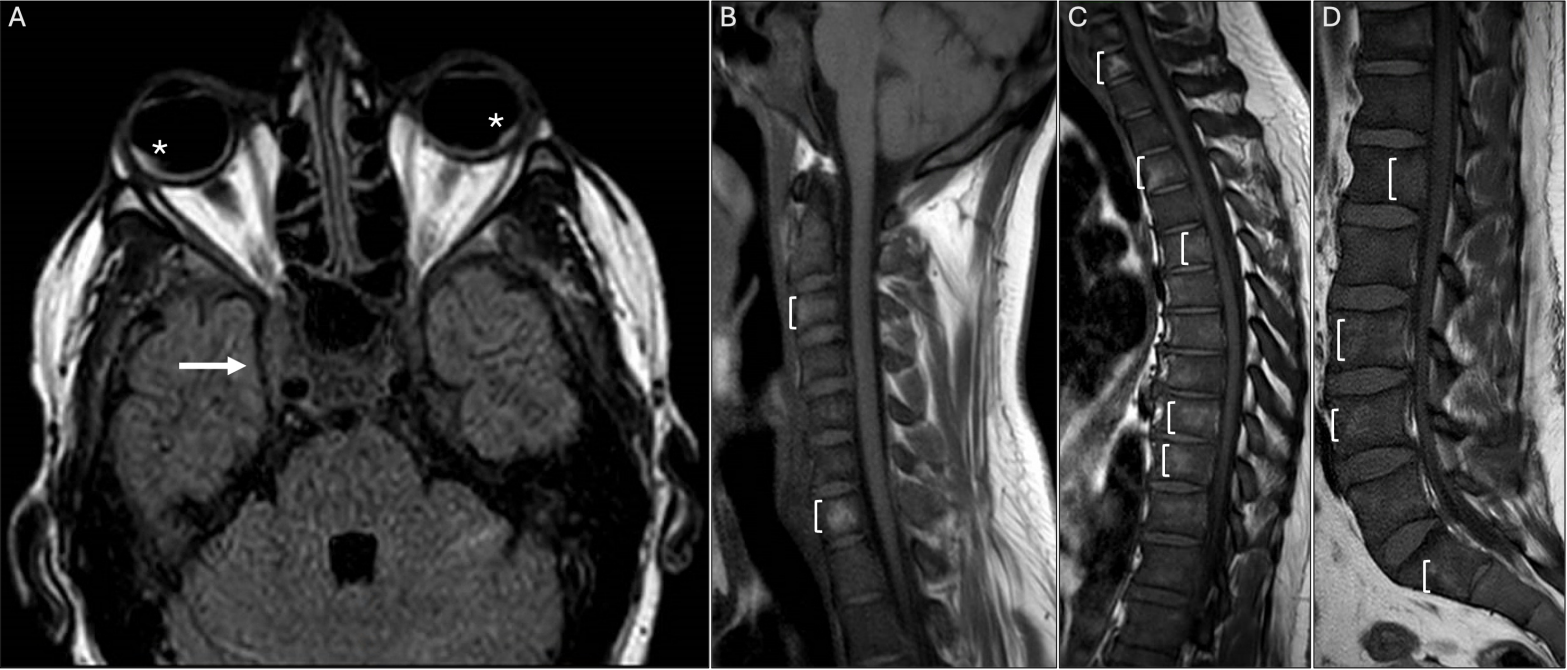
**(A)** Brain T2-weighted fluid-attenuated inversion recovery axial magnetic resonance image (MRI) demonstrates right-sided bulging of the cavernous sinus (arrow) and bilateral posterior intraocular hyperintensities corresponding to SRD (asterisk). **(B-D)** T1-weighted MRI shows heterogeneous bone marrow signal throughout the spine, involving the posterior elements, with diffuse low signal intensity on T1 (brackets); and high signal on T2, with patchy contrast enhancement (not shown).

During his inpatient stay, acute progression was observed at the 3-day follow-up evaluation, with the patient developing complete ophthalmoplegia and full ptosis of the right eye (**Figure 1B**). Visual function remained unchanged, and clinical findings were stable in the left eye. Chest radiography showed unremarkable results. Computed tomography (CT) angiography demonstrated no evidence of vasculitis or aneurysm. Magnetic resonance imaging (MRI) of the brain and orbits showed a right-sided prominence of the cavernous sinus and bilateral posterior intraocular hyperintensities corresponding to SRD, without evidence of orbital or periocular lesions (**Figure 2A**), and magnetic resonance venography excluded sinus thrombosis or stenosis.

Lumbar puncture and cerebrospinal fluid (CSF) analysis revealed an opening pressure of 20 cm H_2_O, elevated white blood cell count (25 cells/μL), increased protein concentration (64.9 mg/dL), and normal glucose level (115 mmol/L). Both the venereal disease research laboratory test and cytology showed negative results in CSF. An extensive rheumatologic panel was significant only for decreased total IgG and IgG subclasses (1–4); all other rheumathologic parameters were within normal limits, including HLA-B5701, HLA-B27, rheumatoid factor, complements C3 and C4, beta-2 glycoprotein IgM, IgG, and IgA antibodies, anti-cyclic citrullinated peptide antibody, anti-SS-A and anti-SS-B IgG, cytoplasmic and perinuclear anti-neutrophil cytoplasmic antibodies, lupus anticoagulant, angiotensin-converting enzyme, myelin oligodendrocyte glycoprotein antibody, and anti-aquaporin 4 antibody.

Additionally, neoplastic workups, such as serum protein electrophoresis, prostate-specific antigen, and fecal occult blood testing, showed unremarkable findings. Infectious disease testing showed a similarly negative result, including HIV-1 antigen and antibody, HIV-2 antibody, hepatitis profile, chlamydia, rapid plasma reagin, fluorescent treponemal antibody absorption, and QuantiFERON-TB Gold. After excluding an acute infectious process, the patient received methylprednisolone 1.0 g IV daily for 3 days, with no clinical improvement. Due to persistent back pain, a spinal MRI was performed, revealing pathological changes consistent with an infiltrative process throughout the spine (**Figures 2B–D**). Staging with thoracic and abdominopelvic CT scans revealed splenomegaly, multiple retroperitoneal lymph nodes, and a hypodense hepatic lesion, suggestive of a lymphoproliferative disorder.

**Figure 3:**
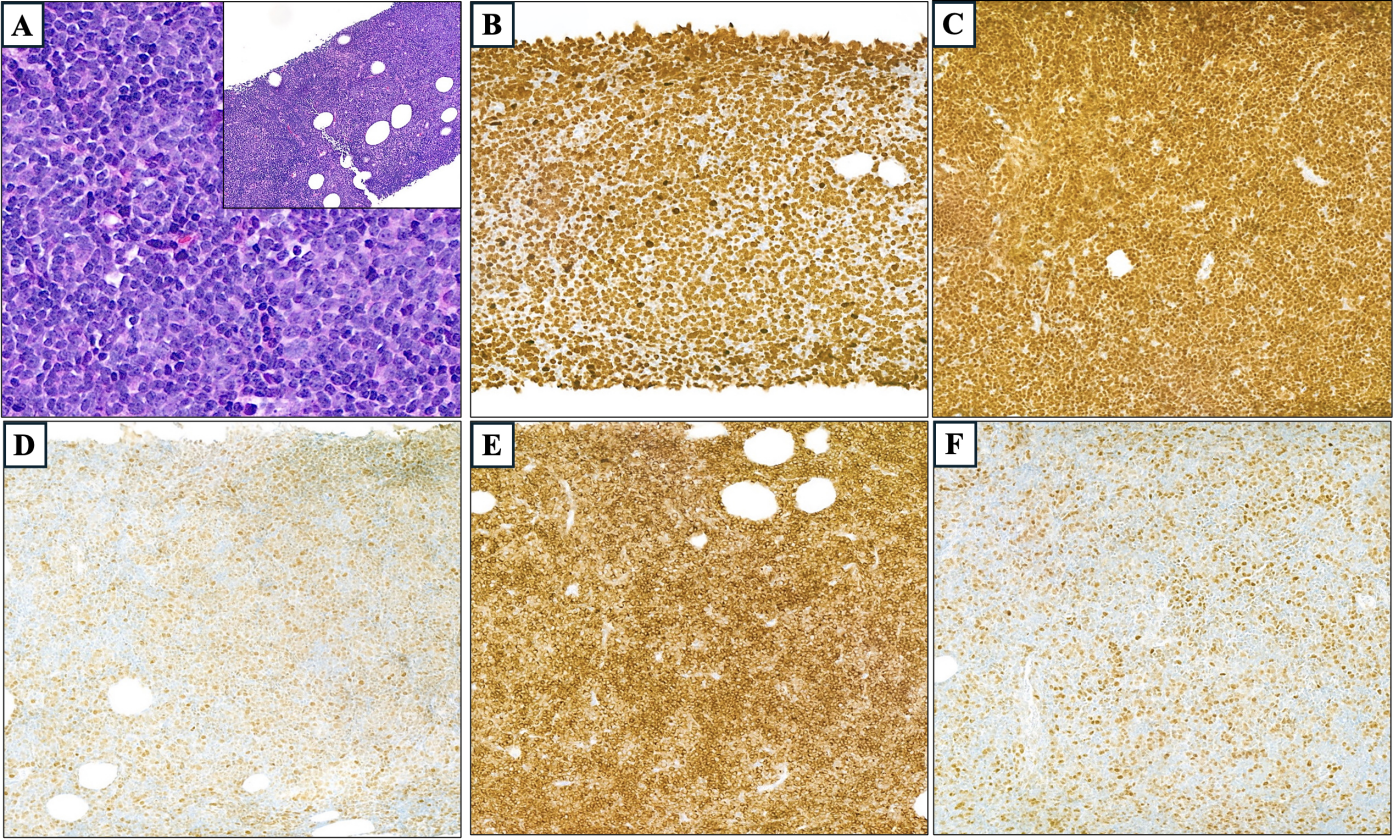
The histopathologic appearance of the biopsy specimen shows that the infiltrate is composed of large atypical lymphoid cells with vesicular chromatin and prominent nucleoli (magnification ×60). Inset: effacement of normal lymphoid architecture and replacement by a diffuse infiltrate (magnification ×10); stain hematoxylin and eosin **(A)**. In immunohistochemistry sections, Ki67 showed a proliferative index of nearly 100% **(B)**, strong and diffuse PAX-5 positivity **(C)**, Bcl-6 positivity in more than 30% **(D)**, Bcl-2 positivity in more than 50% **(E)**, and c-Myc positivity in more than 40% **(F)**, magnification ×20.

Subsequently, a CT-guided biopsy of the mesenteric lymph node was performed. Morphologic and immunophenotypic analyses confirmed the diagnosis of DLBCL, germinal center type by cell of origin (**Figures 3A–C**), with Bcl6, Bcl2, and c-Myc rearrangements (triple-hit) (**Figures 3D–F**, respectively). Flow cytometric analysis of the CSF demonstrated CD10-positive B-cell lymphoma (comprising 84% of total cells, lambda light chain restricted). Given the extent of disease and CNS involvement, the patient was treated with intrathecal chemotherapy (methotrexate, hydrocortisone, cytarabine) and systemic chemotherapy using the TEDDi-R regimen (temozolomide, etoposide, doxil, dexamethasone, ibrutinib, and rituximab).

**Figure 4:**
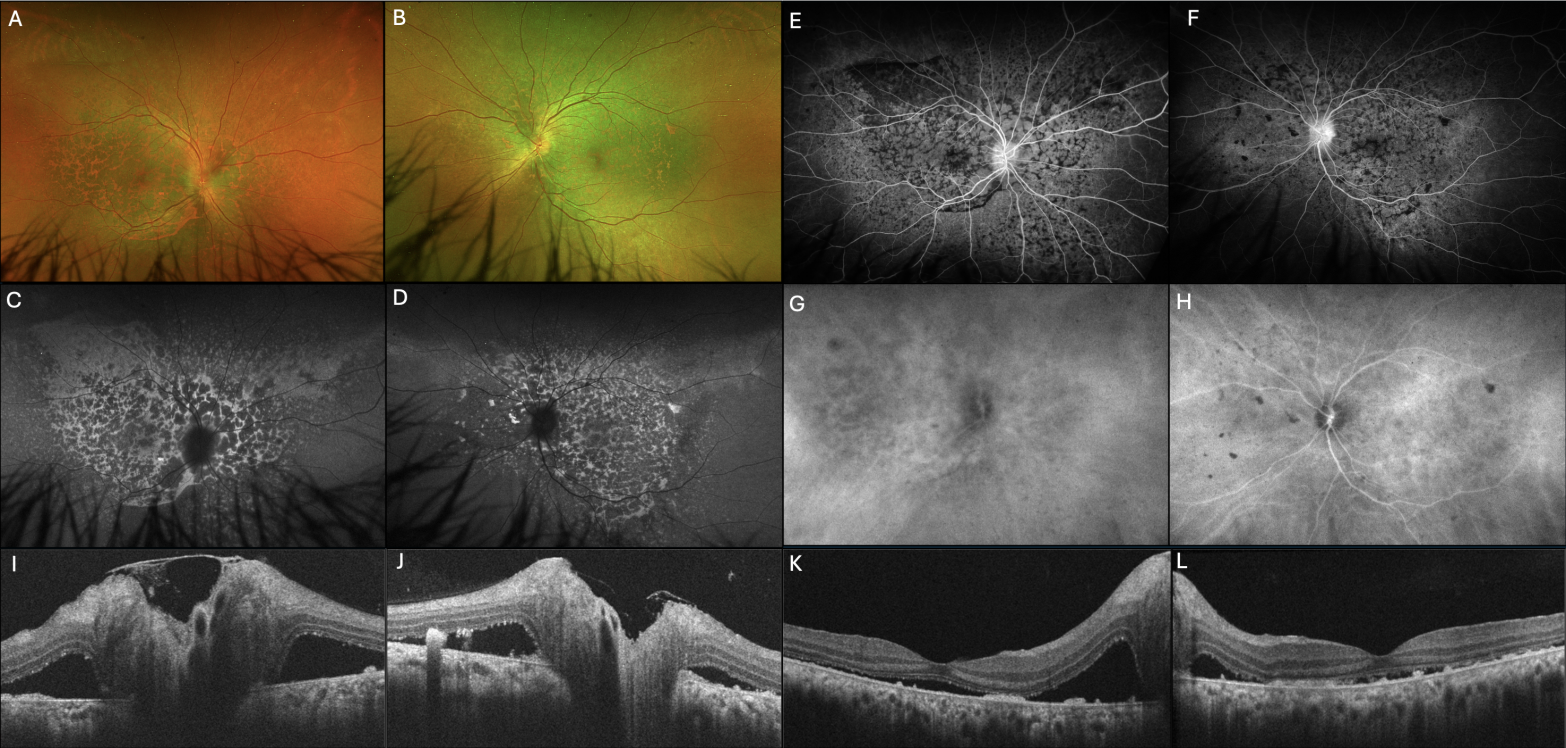
**(A, B)** Fundus photographs demonstrate improvement of SRD with persistent patchy areas of hypo- and hyperpigmentation. **(C, D)** Fluorescein autofluorescence (FAF) shows patchy areas of hypo-autofluorescence surrounded by zones of increased autofluorescence in a leopard-spot pattern. **(E, F)** Fluorescein angiography (FA) shows hyper-fluorescent patches surrounded by zones of hypo-fluorescence, an inverse FAF pattern. **(G, H)** Indocyanine green angiography (ICGA) demonstrates focal hypocyanescent areas in the posterior pole of both eyes. Optical coherence tomography of the optic nerve **(I, J)** and macula **(K, L)** shows residual subretinal fluid in the macula and peripapillary regions. Focal areas of retinal pigment epithelium irregularities with hyperreflective subretinal depositions are seen in both eyes.

After 2 months of treatment, the patient demonstrated marked improvement, with a visual acuity of 20/40 in the right eye and 20/25 in the left eye with refractions of +0.50 −0.50 at 10° and +0.50 −0.25 at 75°, respectively. In addition, EOM were full and symmetric in all gazes, and there was resolution of ptosis. Posterior segment examination showed persistent patchy pigmentary changes and improvement of SRD (**Figures 4A, B**), confirmed by OCT (**Figures 4I–L**) and B-scan ultrasonography. Post-treatment ultrasonography also demonstrated resolution of choroidal thickening. FAF showed scattered mottled areas of hypo-autofluorescence surrounded by areas of hyper-autofluorescence in a leopard-spot pattern (**Figures 4C, D**). FA was remarkable for hyper-fluorescent patches surrounded by zones of hypo-fluorescence, an inverse FAF pattern (**Figures 4E, F**). ICGA demonstrated focal areas of choroidal hypocyanescence in the posterior pole of both eyes (**Figures 4G, H**).

## Discussion

Secondary DLBCL affecting the eye is rare and most frequently infiltrates the choroid (12). In cases of uveal metastasis, the extensive vascular supply of the choroid allows hematogenous spread of lymphomas, particularly affecting the posterior pole (13, 14). Clinical features of secondary choroidal lymphoma include ocular pain, severe decrease in visual acuity, vitreous reaction, or media haziness (6). The present case describes an atypical presentation of DLBCL, characterized by ophthalmoplegia, bilateral SRD, and choroidal thickening.

Fukutsu et al. described a case of choroidal lymphoma presenting with bilateral SRD and imaging features that resembled pseudo-inflammatory manifestations of VKH disease (15). Additionally, the combination of oral numbness, along with painful ophthalmoplegia, has been linked to hematologic malignancies, including DLBCL (16, 17). Mandibular symptoms may be an early sign of neoplastic infiltration of the mental canal (16, 18, 19), while the ophthalmoplegia may result from direct compression or infiltration of adjacent orbital structures (16, 20, 21).

In our case, the patient exhibited a combination of the previously described manifestations, highlighting the diverse presentations and the complexity of diagnosis. The complete ophthalmoplegia with an associated non-reactive pupil and normal afferent pupillary response suggested concurrent palsy of cranial nerves 3, 4, and 6. Anatomically, lesions involving the orbital apex, superior orbital fissure, or cavernous sinus are generally considered. However, involvement of the mandibular nerve, which traverses through the foramen ovale and not the cavernous sinus, may be indicative of multifocal lesions such as a neoplastic infiltrative process, rather than a single tumor.

Subtypes of DLBCL have been established based on molecular analysis of genes such as c-Myc, Bcl2, and Bcl6 (22). Lymphomas with genetic rearrangements involving all three genes are termed triple-hit. This subtype is classified as a high-grade lymphoma, is associated with significantly poorer prognosis, and requires more intensive chemotherapy regimens (23). Recent studies have reported superior outcomes in patients treated with R-EPOCH (rituximab, etoposide, prednisone, vincristine, cyclophosphamide, and doxorubicin) compared to traditional R-CHOP (rituximab, cyclophosphamide, doxorubicin, vincristine, prednisone), with a significantly longer overall survival and a reduction in the risk of death of up to 50% (23). However, prospective data on optimal treatment remain limited (22). In our case, the patient demonstrated significant clinical improvement in both systemic and ocular symptoms following combined intrathecal and systemic chemotherapy. However, follow-up of our patient was limited, and long-term prognosis may be uncertain.

Involvement of the CNS in patients with DLBCL is uncommon, with an estimated incidence of 2%–5% (24–27). Nonetheless, according to the CNS International Prognostic Index, the presence of risk factors such as elevated LDH, metastasis to multiple organs, and advanced disease staging has been associated with an increased risk of CNS involvement or relapse (28). Secondary CNS disease confers a poor prognosis, with reports estimating an overall survival ranging from 3 to 22 months after diagnosis (27, 29, 30).

At presentation, secondary choroidal DLBCL is a comparatively high-grade lymphoma, characterized by a more rapidly progressive course and a higher rate of poor vision than primary cases (3, 5, 6, 11). Nonetheless, visual function after treatment generally improves or remains stable (6). Similarly, our patient had significant clinical improvement in visual acuity and extraocular movements, with resolution of ptosis. Also, decreases in choroidal thickness and SRD sizes were observed following chemotherapy.

Identifying the etiology of the ocular findings in our patient was challenging, especially in the absence of a previous history of systemic malignancy. Patients with secondary choroidal DLBCL generally have severe vision loss and may be systemically ill, which hinders the acquisition of ancillary imaging and poses additional diagnostic challenges. The presence of concomitant multiple cranial nerve palsies and SRD associated with increased choroidal thickness along with systemic signs and symptoms (weight loss and chronic back pain) in patients with risk factors (smoking) and altered laboratory workup (elevated ESR, CRP, and LDH) suggests a systemic process rather than a localized ocular disease and may promote the involvement of additional primary services in the management of patients. Moreover, neurological imaging and ancillary tests such as OCT, B-scan, FA, and ICG are useful for further characterizing the pathophysiology and guiding systemic workup and treatment, particularly in the presence of atypical ocular findings.

## Conclusions

Choroidal DLBCL secondary to metastasis is uncommon, and its clinical presentation may vary depending on the extent of the disease. Moreover, various imaging modalities are often required; still, delayed or misdiagnosis is not uncommon (6). This case underscores the importance of considering secondary lymphoma in patients presenting with bilateral SRD and neuro-ophthalmic deficits, even in the absence of known systemic malignancy. The combination of cavernous sinus syndrome with concomitant mandibular nerve involvement should prompt CNS and systemic evaluation for hematologic malignancy. Multimodal imaging is critical for diagnosis and post-chemotherapy reevaluation. Patients with triple-hit DLBCL phenotype may achieve dramatic visual recovery following modern targeted chemoimmunotherapy.

## Data Availability

The original contributions presented in the study are included in the article/supplementary material. Further inquiries can be directed to the corresponding author.
